# Mining, visualizing and comparing multidimensional biomolecular data using the Genomics Data Miner (GMine) Web-Server

**DOI:** 10.1038/srep38178

**Published:** 2016-12-06

**Authors:** Carla Proietti, Martha Zakrzewski, Thomas S. Watkins, Bernard Berger, Shihab Hasan, Champa N. Ratnatunga, Marie-Jo Brion, Peter D. Crompton, John J. Miles, Denise L. Doolan, Lutz Krause

**Affiliations:** 1 QIMR Berghofer Medical Research Institute, Brisbane, QLD, Australia; 2 Nestlé Research Centre, Vers-chez-les-Blanc, Lausanne, Switzerland; 3 The University of Queensland Diamantina Institute, The University of Queensland, Translational Research Institute, Brisbane, QLD, Australia; 4 Malaria Infection Biology and Immunity Unit, Laboratory of Immunogenetics, National Institute of Allergy and Infectious Diseases, National Institutes of Health, Rockville, Maryland 20852, USA; 5 Centre for Biosecurity and Tropical Infectious Diseases, Australian Institute of Tropical Health & Medicine, James Cook University, Cairns, QLD, Australia

## Abstract

Genomics Data Miner (GMine) is a user-friendly online software that allows non-experts to mine, cluster and compare multidimensional biomolecular datasets. Various powerful visualization techniques are provided, generating high quality figures that can be directly incorporated into scientific publications. Robust and comprehensive analyses are provided via a broad range of data-mining techniques, including univariate and multivariate statistical analysis, supervised learning, correlation networks, clustering and multivariable regression. The software has a focus on multivariate techniques, which can attribute variance in the measurements to multiple explanatory variables and confounders. Various normalization methods are provided. Extensive help pages and a tutorial are available via a wiki server. Using GMine we reanalyzed proteome microarray data of host antibody response against *Plasmodium falciparum*. Our results support the hypothesis that immunity to malaria is a higher-order phenomenon related to a pattern of responses and not attributable to any single antigen. We also analyzed gene expression across resting and activated T cells, identifying many immune-related genes with differential expression. This highlights both the plasticity of T cells and the operation of a hardwired activation program. These application examples demonstrate that GMine facilitates an accurate and in-depth analysis of complex molecular datasets, including genomics, transcriptomics and proteomics data.

The development of high-throughput molecular assays has revolutionized medical and biomolecular research and has greatly advanced genome, transcriptome and proteome analysis. Researchers are now able to rapidly and cost-effectively profile gene transcription, protein abundances, immune responses, DNA methylation, histone occupancy, microRNA expression, or metagenomic gene frequencies. Measurements are frequently obtained for hundreds to thousands of features and across multiple samples, biological conditions and time-points. Common characteristics of the generated data are their complexity, multidimensionality and multivariate nature, where variance in the measurements can be attributed to multiple explanatory variables and possible confounders. Analysis of the generated data therefore requires the application of advanced data-mining techniques, multivariate statistics and powerful visualization methods for data exploration.

Several computational tools are available for the analysis of complex biomolecular datasets, such as data generated by protein microarrays or NanoString expression data. The Cyber-T web server^1^ allows data transformation and data comparison via Bayesian t-test and ANOVA. The standalone MeV MultiExperiment Viewer^2^ provides powerful methods for data visualization and clustering. Matrix2png^3^ allows visualization of microarray data and other data types via a heatmap. Additionally, a multitude of R packages are available for the analysis of protein microarrays and other biomolecular data. However, thorough and meaningful analysis of complex biomolecular data using these currently available tools is frequently hampered by the advanced computational skills generally required to use them, by their limited selection of statistical methods, or by the lack of visualization capabilities.

To address this, we developed GMine, a powerful, yet easy to use tool for the higher-level analysis of biomolecular data. GMine allows the analysis of any genomic, metagenomic, transcriptomic or proteomic dataset with several hundred to several thousand biological features (e.g. genes or proteins) across different samples and biological conditions. Measurements can represent intensities, frequencies or counts (e.g. signal intensities, expression values, methylation levels, or sequence read counts generated in metagenomic, ChIP-seq or RNA-seq experiments). The software has been developed with an initial focus on protein microarray antibody responses and NanoString gene expression data. These two techniques are becoming widely used in biomolecular and medical research, but there is a general lack of available easy-to-use software for the analysis of the generated data.

Whole-proteome microarrays are increasingly popular for vaccine target antigen identification, studying immune responses, biomarker discovery, and clinical diagnostics^4^,^5^,^6^,^7^,^8^,^9^,^10^. In the field of infectious diseases, protein microarrays are widely used for measuring host immune response to proteins of infectious agents, including malaria, schistosomes or viruses^4^,^5^,^11^,^12^,^13^,^14^,^15^. Protein microarrays allow measurement of antibody responses against the entire proteome of a pathogen. This results in large and complex datasets, with signal intensities measured for thousands of proteins and hundreds of samples. In addition, antibody responses can be attributed to multiple covariates, including gender, age, geographic location, or infection history. The analysis of this type of data therefore requires advanced statistical methods that can assess complex associations while accounting for the various contributing confounding variables; this is enabled by GMine.

NanoString technology performs multiplex quantification of gene expression with high levels of precision and sensitivity, and can detect very small changes of a single transcript. Given the capacity to directly ‘count’ molecules, the platform is free from the inherent multiplex/PCR bias seen in other technologies, such as qPCR, microarray or RNAseq. NanoString enables the precise quantification of expression levels of a large number of genes across multiple biological samples and changes in gene expression can usually be attributed to multiple explanatory variables and confounding factors. The analysis of the generated high-dimensional and multivariate data therefore requires the application of advanced data-mining methods, which is now possible via the GMine web-server.

To demonstrate the utility of GMine for proteome and expression data, we applied our software to two types of studies. First, we used GMine to further analyze previously published proteome microarray data generated in a prospective study investigating host immune response to the malaria parasite *Plasmodium falciparum*^15^. Our intent was to apply GMine to this existing dataset to identify *P. falciparum* antigens associated with protection from clinical malaria, in order to facilitate the rational design of malaria vaccines. As a second applied study, we used GMine to analyze gene transcription occurring during the course of T cell activation, by comparing the expression of 135 key genes in resting and activated primary human CD4^+^ and CD8^+^ T cells. These two T cell subtypes play crucial roles in orchestrating and executing human immune system function, and understanding their molecular basis is important for guiding and modulating function for preventive or therapeutic purposes.

## Results

### GMine Server and User Interface

Genomics Data Miner (GMine) is accessible via a free web-server, which provides a user-friendly interface to advanced statistical methods and data-mining algorithms (Table 1). The web-frontend is implemented in Java using the JavaServer Faces architecture and the backend is implemented in Perl and the R statistical programming environment. The user interface is based on HTML and Cascading Style Sheets and interactive visualizations have been realized using JavaScript. No installation, configuration, registration or login is required. Data is kept privately and cannot be viewed by other users. Uploaded data and calculated results are deleted after a user session has terminated. Extensive help pages and a tutorial are provided via a wiki server. GMine is freely available at http://cgenome.net/GMine/.

The web-frontend provides several analysis tabs that provide access to different data-mining methods (Table 2). High-quality figures are generated in PNG, PDF or SVG format, which can readily be used across an array of scientific outputs. The SVG format allows an easy modification of figures in vector graphics editors, such as Inkscape. Generated figures can be adjusted according to user preferences, including color scheme, coloring of samples by biological condition, figure resolution and figure dimensions.

### Data Upload and Normalization

As input, GMine requires an ‘n × m’ data matrix and a metadata file providing meta-information for each sample (Table S1). The server can be used for any n × m data matrix (with n × m < two million), where the columns correspond to samples, the rows represent features (e.g. proteins) and the values of the data matrix represent measured intensities, frequencies or counts (e.g. signal intensities, expression values, or methylation levels). Input files are simple text files in tab separated or comma separated format and can be created in spreadsheet programs such as Excel. The data upload page provides several normalization and data transformation methods, including quantile normalization, centered log ratio and variance stabilization and calibration for microarray data (VSN) (Table 1). The effects of transformation and normalization procedures on the data are visualized by boxplots and scatterplots (Figure S1). The provided VSN algorithm was originally developed for gene expression microarrays, and is now also widely used for the normalization of protein array data. VSN corrects for the dependency between intensity and variance, which can bias the analysis in standard statistical procedures. The algorithm transforms the data such that the variance remains nearly constant over the whole intensity spectrum.

### Analysis Enabled by GMine

#### Quantitative visualization and univariate data analysis

**Figure 1:**
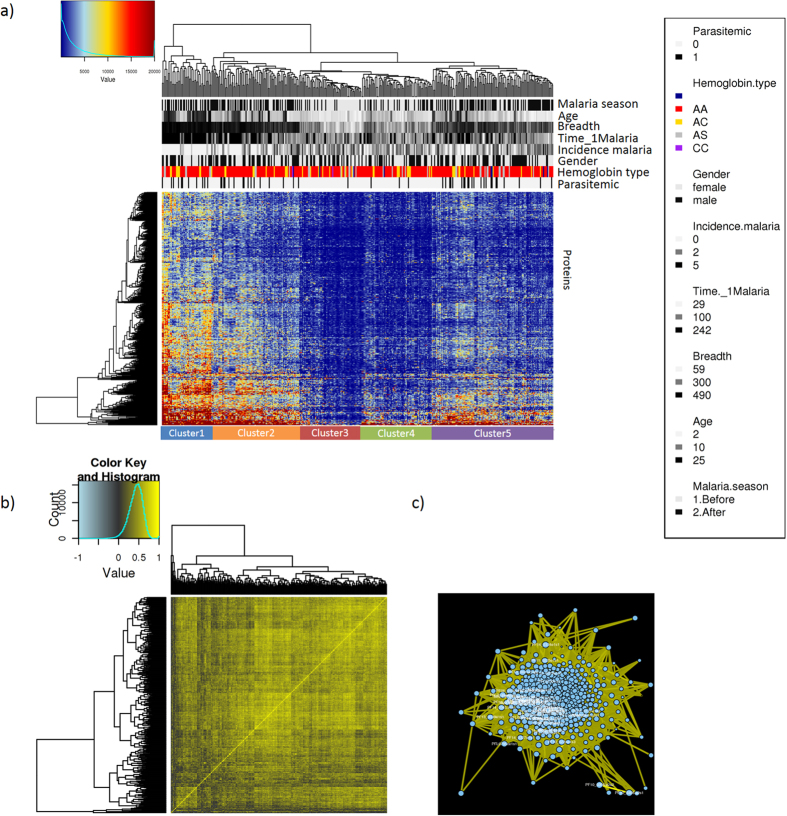
Antibody response to 491 seropositive malaria parasite (*P. falciparum*) proteins. From each individual (n = 194), two samples were included (one before and one after the malaria season). (**a**) Hierarchical clustering of antibody signal intensities of 491 seropositive *P. falciparum* proteins. Proteins were considered seropositive for antibody response if the signal intensity was greater than the mean signal intensity of No-DNA negative controls plus 1.5 times the standard deviation (SD). Antibody response to *P. falciparum* proteins form five distinct clusters associated with subject age, time of the malaria season and incidence of clinical malaria. Cluster 1 represented samples collected after the malaria season from adults who were parasite positive and showed low incidence of malaria during the 8 months study period. Cluster 2 represented antibody profiles collected before the malaria season of older children, parasitemic with higher incidence of clinical malaria. Cluster 3 represented antibody profiles before the malaria season of young children, who were non parasitemic with high incidence of clinical malaria during the study period. Cluster 4 was associated with antibody profiles after the malaria season of older children who had high incidence of clinical malaria. Cluster 5 represented antibody profiles after malaria season of older children, who were parasitemic and had a low incidence of clinical malaria. (**b**) Heatmap of Pearson’s correlations between signal intensity of 491 seropositve proteins. (**c**) Network analysis of host antibody response against *P. falciparum* proteins. Nodes represent parasite proteins, yellow and blue edges represent positive and negative correlations, respectivley.

**Figure 2:**
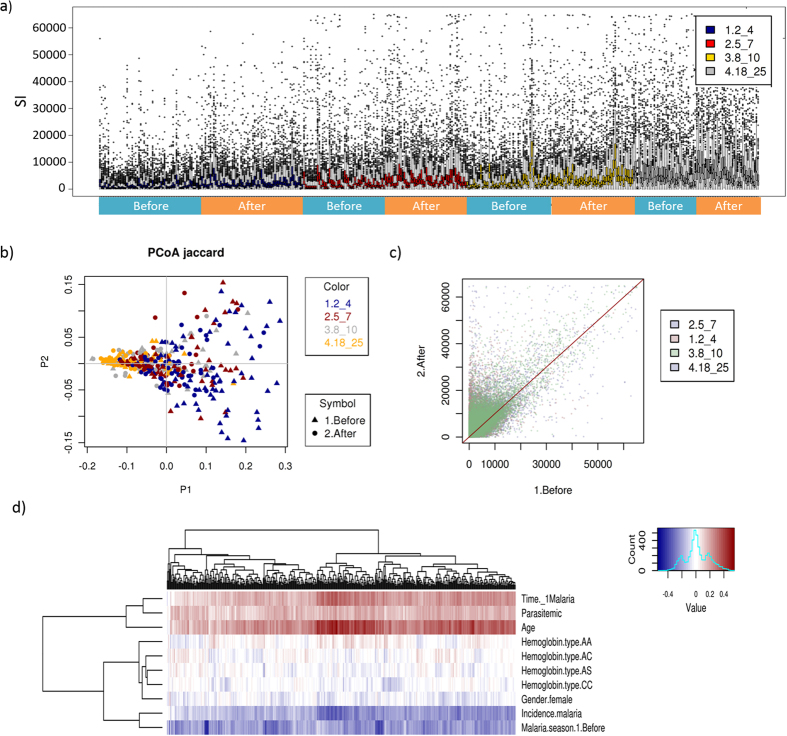
Antibody reactivity increased with age and from before to after the malaria season (n = 194 individuals, two samples per individual). (**a**) Box plot showing the signal intensities of 491 seropositive proteins before and after the malaria season in each age group (2–4, 5–7, 8–10 and 18–25 years). (**b**) Principal coordinates analysis (PCoA) of antibody signal intensities (Jaccard distance). Principal coordinate 1 explained variation in the antibody reactivity associated with age, principal coordinate 2 the variation attributed to the time of the malaria season (before or after). Colors indicate age group and symbols the time of the malaria season. Individuals cluster by age and time of the malaria season. (**c**) Scatter plot of signal intensities of 491 seropositive proteins before and after the malaria season. Each dot represents one protein. Dots are significantly shifted towards the upper left, indicating that antibody response against *Plasmodium* proteins is generally stronger after the malaria season (y-axis). This shift is significant for each of the four age groups (p < 2.9 × 10^−62^ for all age groups). (**d**) Heatmap visualizing Pearson’s correlation between antibody signal intensities, age, concurrent parasitemia, gender, hemoglobin type, time during the malaria season, incidence of malaria episode and days until the first malaria episode. Positive correlations are shown in red, negative correlations in blue.

**Figure 3:**
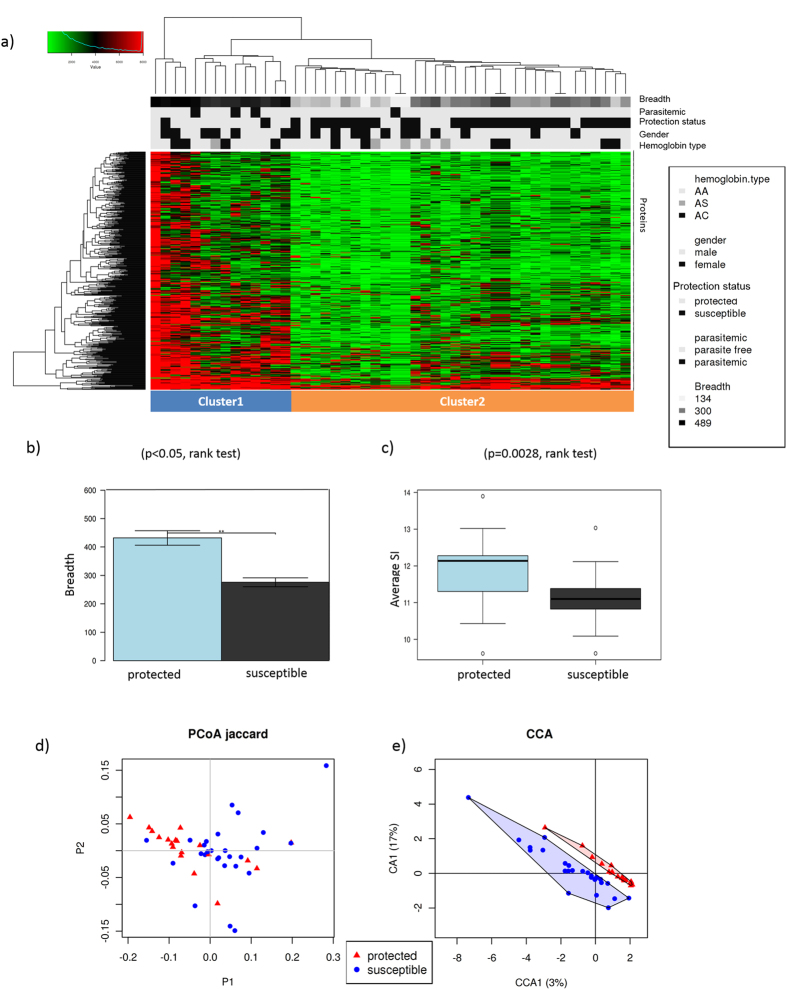
*Plasmodium*-specific antibody profiles in protected and susceptible children. Children of 8–10 years of age (n = 48) were defined “protected” if they did not experience a clinical malaria episode during the 8-month study period (n = 19) and “susceptible” if they did experience ≥1 malaria episodes (n = 29 children). Antibody response was measured at the beginning of the 8-month study period before the malaria season. (**a**) Heatmap of the antibody signal intensities against 491 seropositive *P. falciparum* proteins for the 48 children. Antibody response to parasite proteins showed a marked difference between protected (n = 19) and susceptible (n = 29) children. Protected children (Cluster 1) had a significantly higher breadth and magnitude of antibody response and responded to a different combination of proteins compared to susceptible children (Cluster 2). Breadth of antibody responses (number of seropositive *Plasmodium* proteins) for each individual (**b**) and magnitude of antibody responses (average signal intensity of seropositive proteins) (**c**) in protected and susceptible children. Significance of differences in breadth and magnitude of antibody response was measured by Wilcoxon–Mann–Whitney test. (**d**) Principal coordinate analysis (PCoA) of antibody signal intensities before the malaria season of the 491 seropositive proteins (Jaccard distance). Children clustered by protection status (protected versus susceptible). (**e**) Canonical correspondence analysis (CCA) of antibody signal intensities including protection from malaria (protected/susceptible), gender, concurrent parasitemia and hemoglobin type as explanatory variables. Immunity to malaria was significantly associated with antibody response (CCA p = 0.020), while gender (CCA p = 0.332), parasitemia (CCA p = 0.162) and hemoglobin type (CCA p = 0.329) were not.

**Figure 4:**
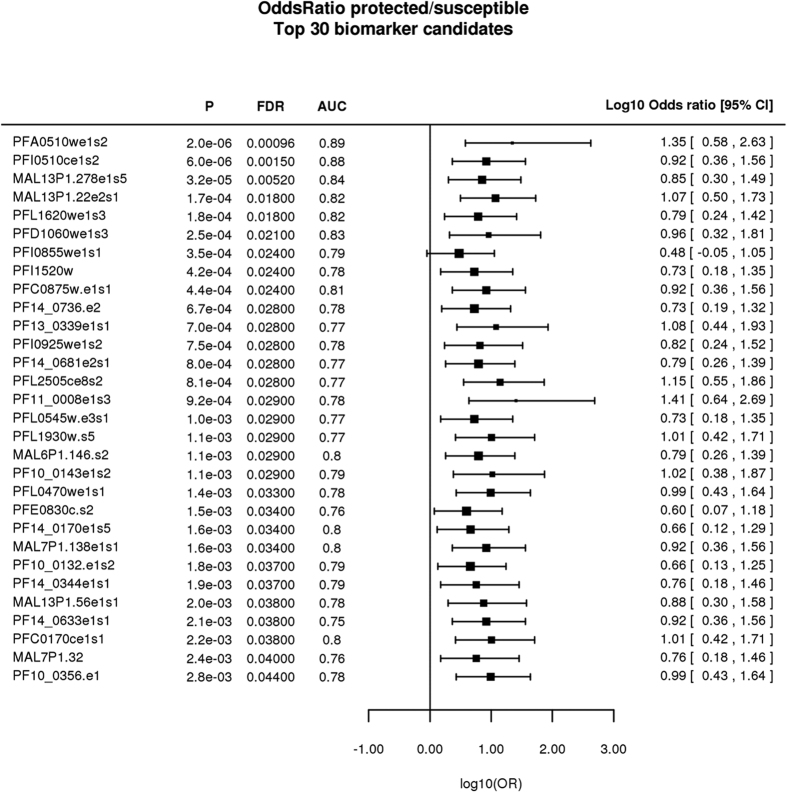
Proteins predictive of protection from clinical malaria in children between 8–10 years old children (n = 48). We examined the power of antibody signal intensities at baseline (before malaria season) to predict protection from malaria during the eight months malaria period. Signal intensities between protected and susceptible children were compared by t-test and p-values were adjusted for multiple testing by false discovery rate (FDR). We identified 62 proteins significantly associated with protection (FDR < 0.05). The forest plot presents p-values, FDR, area under the curve (AUC) and odds ratio (OR) for the association between antibody response at baseline and subsequent protection during the 8 months malaria season. Only the top 30 most significant proteins are shown. The AUC describes the discriminatory power of each protein to distinguish protected children from susceptible children. AUC values ≥0.8 indicate a strong discriminatory power.

**Figure 5:**
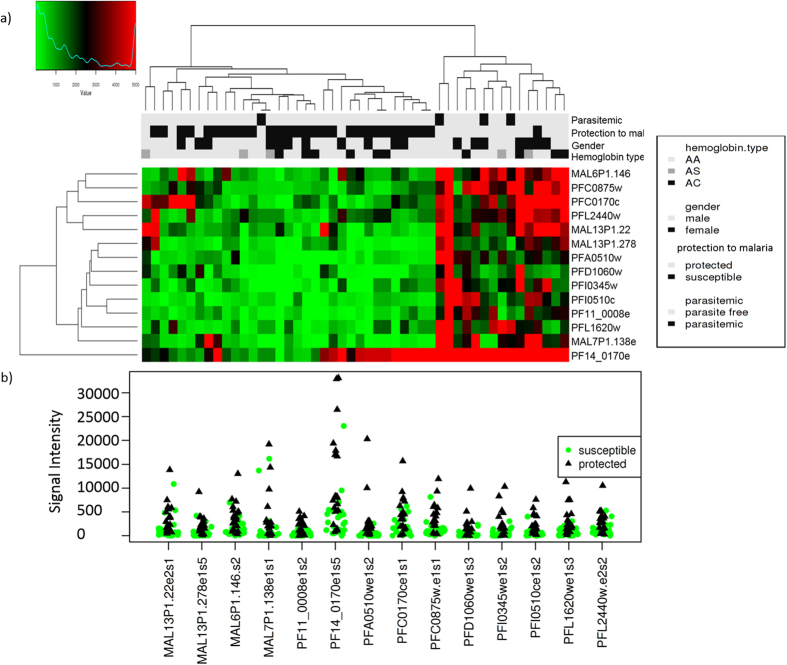
Antibody response against fourteen *P. falciparum* proteins found to discriminate between protected (n = 19) and susceptible (n = 29) children with high accuracy (AUC range = 0.8–0.9). (**a**) Heatmap of antibody reactivity measured at baseline before the malaria season. Antibody reactivity profiles of protected and suceptible children form distinct clusteres. (**b**) Stripchart representing the antibody signal intensities in protected and susceptible children.

**Figure 6:**
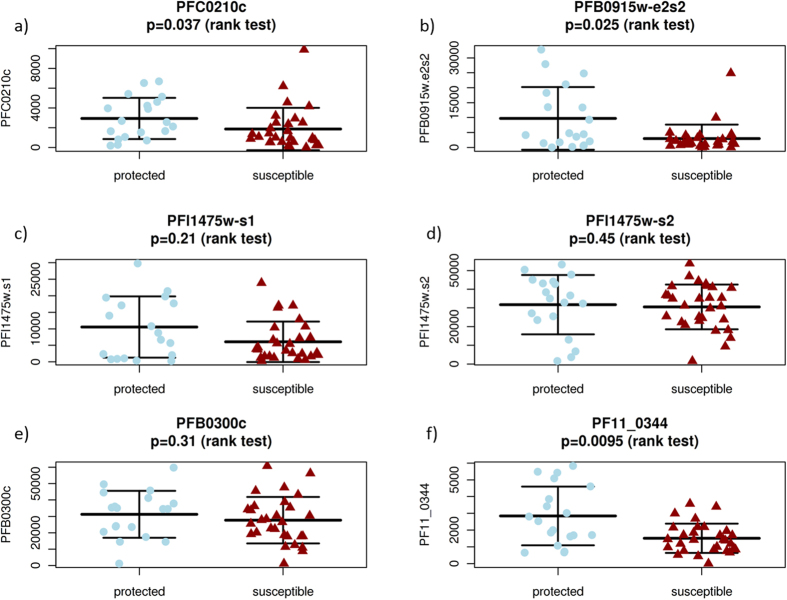
Antibody response against the leading malaria vaccine candidates included in the protein microarray in protected and suceptible children. Antibody reactivity against circumsporozoite protein (CSP, PFC0210c) (**a**), liver stage antigen 3 (LSA3, PFB0915w) (**b**), merozoite surface protein 1 (MSP-1, PFI1475w) (**c**,**d**), MSP-2 (PFB0300c) (**e**) and apical membrane antigen 1 (AMA-1, PF11_0344), did not discriminate between protected and susceptible children (FDR > 0.05). P-value from rank test is shown.

**Figure 7:**
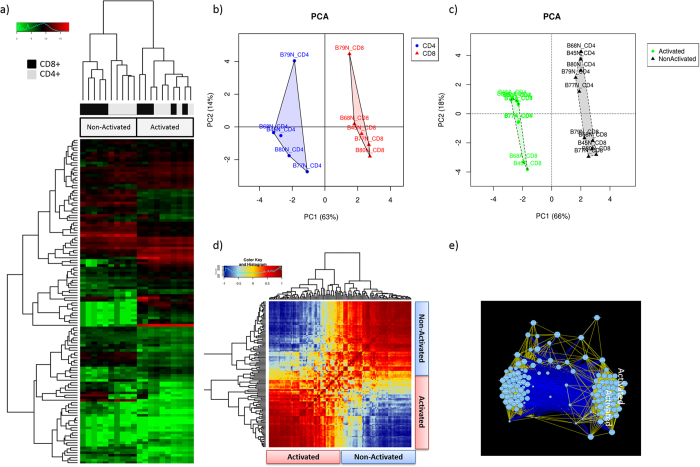
Unsupervised clustering of different human T cell lineages at rest (non-activated) and post activation state. Primary and activated CD4^+^ and CD8^+^ T cells were sorted from 5 healthy individuals and profiled on a custom 135-plex nCounter codeset designed around key genes for T cell phenotyping and function. Hierarchical clustering (**a**) and PCA (**b**,**c**) showed clear clustering of gene expression profiles by T cell subtype (CD4^+/^CD8^+^) in resting state (**b**) and T cell activation status (activated/non-activated) (**c**). PCA was coloured by T cell subtype (**b**) and activation status (**c**). Correlation of CD4^+^ gene expression levels presented as heatmap (**d**) and network (**e**). Correlation of CD4^+^ gene expression levels was measured by Pearson’s correlation. The heatmap presents correlations by colour code, ranging from blue (negative association) to red (positive correlation). The network depicts genes as nodes, positive correlations by yellow edges and negative correlations by blue edges. The heatmap and the network analysis showed two distinct, highly correlating gene clusters, representing genes with high expression in activated or non-activated T cells, respectively.

Data is visualized quantitatively using heatmaps, bubble plots, scatter plots, stripcharts, barcharts and boxplots (Figs 1, 2, 3, 4, 5, 6 and 7). Biological conditions or classes (e.g. case/control) can be compared with a wide range of parametric and non-parametric statistical tests (Table 1) and p-values are adjusted for multiple testing by FDR and Bonferroni correction. Distribution of p-values is presented as histograms and quantile-quantile (QQ) plots. QQ plots characterize the extent to which the observed distribution of the test statistics follows the expected (null) distribution. This allows the detection of evidence for systematic bias. GMine also facilitates hypothesis testing by the Bayesian t-test, which addresses problems associated with low replication levels and technology biases and has been proposed for the analysis of DNA microarrays, protein arrays, quantitative mass spectrometry and next-generation sequencing (RNA-seq) data^1^,^16^.

#### Multivariate analysis

Multivariate statistics are powerful techniques that can identify complex associations between measurements and multiple explanatory variables. GMine provides a wide range of unsupervised multivariate methods for ordination and clustering (Table 1). When visualizing data in heatmaps (Figs 1a, 3a, 5a and 7a), GMine allows trimming of outliers, selection of the color palette and adjustment of the color range center.

The significance of associations between measurements and multiple explanatory variables can be examined using the supervised statistical methods redundancy analysis (RDA), canonical correspondence analysis (CCA), and multivariable regression. GMine further enables data analysis by Partial Least Squares Regression (PLS), a multivariate methods which can identify associations between individual features and multiple explanatory variables.

Correlation networks visualize positive and negative associations between features, between explanatory variables, and between features and explanatory variables (Figs 1c and 7e). Features and explanatory variables are represented as nodes and measures as node sizes. Edges represent positive (yellow) and negative (blue) associations. Nodes are ordinated in the network by principal coordinates analysis (PCoA), which places correlating nodes in close proximity and anti-correlating nodes at distant locations.

#### Analysis of repeated measures

GMine allows for the analysis of experimental data from a repeated measures design. For instance, repeated measures are obtained in longitudinal studies or when multiple samples are collected from different tissues of the same subject, e.g. tumor and matched normal samples. In GMine, repeated measures can be analyzed using mixed effect regression models, which incorporate the individual (or animal or cage) as a random effect and explanatory variables (e.g. case/control or treatment) as fixed effects. These models can distinguish between group-specific effects (e.g. average in cases and controls) and subject or cage-specific effects.

#### Biomarker discovery and classification

GMine provides simple yet powerful methods for biomarker discovery. The discriminatory power of the uploaded data to distinguish between two sample groups (e.g. cases/controls) is ascertained using a support vector machine (SVM) evaluated by leave-one-out cross-validation. The discriminatory power of biomarker candidates is described by the area under the curve (AUC), odds ratio, delta (difference in means in units of standard deviation), and fold change (Fig. 4).

#### Identification of relevant features from large multidimensional datasets

The high-dimensionality of biomolecular datasets with far more variables than samples can affect the ability of statistical analysis to extract meaningful information^17^,^18^,^19^,^20^. Furthermore, biomolecular data frequently contains many features that are either redundant or irrelevant, and can thus be removed without much loss of information. Robust statistical techniques are needed that are able to extract biologically informative features differentiating two or more phenotypes from large multidimensional data. To identify the subset of relevant features predictive of an outcome of interest, GMine provides access to the advanced feature selection methods: step-wise linear regression, LASSO regularized regression, random forest and the linear discriminant analysis (LDA) effect size method (LEfSe)^20^. LASSO performs both feature selection and regularization to prevent overfitting. Random forest identifies the subset of most relevant features by constructing a collection of decision trees. Overfitting is avoided by constructing trees incorporating only a random subset of the features. LEfSe identifies features most likely to explain differences between classes by coupling standard tests for statistical significance with additional tests encoding biological consistency and effect relevance^20^. In GMine, selected features are presented as barcharts, where bars depict the importance of each feature (Figures S4 and S7).

As a preprocessing step, GMine also facilitates the reduction of data dimensionality by factor analysis. The main aim of factor analysis is to remove redundant features (i.e. highly correlating features) without much loss of information. Correlating features are described by a lower number of latent variables, called factors. Following factor analysis, the reduced dataset can be analyzed in GMine using the full range of statistical techniques provided.

### Application of GMine for the Analysis of Malaria Protein Microarray Data

Using GMine, we re-examined protein microarray data generated in a prospective, longitudinal study investigating host immune response to the malaria parasite *P. falciparum*^15^. The study cohort included 194 individuals from the malaria endemic Kambila region in Mali. From each participant, blood samples were collected before and after the 8-month malaria season. The analyzed dataset represented host antibody response against 491 seropositive *P. falciparum* proteins profiled for 388 serum samples (194 individuals, 2 samples per individual).

Antibody response to the 491 *P. falciparum* proteins was strongly correlated (Fig. 1a,b). However, despite the high level of correlation, antibody profiles were highly subject-specific and clustered by age, time relative to the malaria season (before/after), concurrent parasitemia, incidence of febrile malaria during the 8-month study period, and days until the first malaria episode (Figs 1a and 2b). Antibody reactivity to 483 *P. falciparum* proteins (98.4% of the 491 seropositive proteins) increased with subject age (Fig. 2a) (ANOVA, FDR < 0.05) and was generally higher after the malaria season (Fig. 2a,c). Children showed a stronger increase in antibody response than adults (Fig. 2a) from before to after the malaria season. The significance of observed associations was tested using the multivariate statistical method redundancy analysis (RDA). In accordance with our clustering results, antibody responses were significantly associated with age, time of sample collection (before or after malaria season) and concurrent parasitemia (p < 0.001) (Figure S2). Age, days until first malaria episode, concurrent parasitemia and time of sample collection at the end of malaria season were positively associated with antibody reactivity; incidence of clinical malaria showed a negative association (Fig. 2d, Figure S2). Gender, hemoglobin type and days until the first episode were not associated with antibody reactivity (RDA, p > 0.05) (Figure S2).

#### Association between antibody response and immunity to malaria

We examined the association between antibody response to *P. falciparum* proteins before the malaria season and subsequent immunity to malaria during the 8-month malaria season. Using multiple linear regression we regressed antibody response on age and immunity to malaria (absence of clinical malaria yes/no during the 8-month season). None of the 491 seropositive *P. falciparum* proteins was association with protection from malaria after correction for multiple testing (Table S2).

#### Association between antibody response and immunity to malaria in children

To avoid the confounding effect of age on malaria risk, we re-analyzed the association between antibody reactivity to *P. falciparum* proteins and subsequent protection against clinical malaria in the subset of children aged 8 to 10 years. For this analysis we compared antibody profiles of children who did not experience clinical malaria during the 8-month malaria season (“protected”, n = 19) versus those who experienced ≥1 malaria episodes (“susceptible”, n = 29).

Protected and susceptible children had remarkably different antibody profiles before the malaria season, with protected children showing higher breadth (number of positive proteins) and stronger reactivity to a wide range of parasite proteins (Cluster 1 in Fig. 3a,b and c). In accordance with our results obtained by unsupervised clustering (Fig. 3a,d), multivariate analysis showed a significant association between antibody response and protection status (p = 0.02, CCA) (Figs 3e and S3). Antibody profiles against *P. falciparum* proteins were highly predictive of immunity to malaria. A support vector machine (SVM) evaluated by leave-one-out cross-validation was able to predict immunity to malaria with 95% accuracy.

We identified 62 parasite proteins with significantly increased mean signal intensity in children with resistance to malaria (FDR < 0.05, t-test) (Table S3). Three of these 62 proteins were highly associated with resistance (Bonferroni corrected p-value < 0.05) and were able to discriminate between protected and susceptible children with high accuracy (AUC of 0.8–0.9) (Table S3, Figs 4 and 5). However, antibody reactivity against proteins representing some of the leading malaria vaccine candidates circumsporozoite protein (CSP, PFC0210c), merozoite surface protein 1 (MSP-1, PFI1475w), merozoite surface protein 2 (MSP-2, PFB0300c), apical membrane antigen 1 (AMA-1, PF11_0344) and liver stage antigen 3 (LSA3, PFB0915w) did not discriminate between protected and susceptible children (FDR > 0.05) (Fig. 6). The high-dimensionality of the gene expression dataset can potentially affect the ability of standard statistical analysis to extract meaningful information. We therefore additionally analyzed the data using the LEfSe algorithm, which identified 12 *Plasmodium* proteins associated with protection (Table S9).

Our results indicate that immunity to malaria is related to a pattern of responses to multiple parasite proteins and not attributable to any single antigen. We therefore sought to identify the signature of the most relevant proteins associated with protection against clinical malaria. Stepwise forward logistic regression identified a six protein signature (Figure S4), which achieved perfect separation (AUC of 1) and improved power for discriminating between protected and susceptible children when compared to individual proteins (Table S3).

### Application of GMine for the Analysis of Gene Expression in Human T Cells Following Activation

Using GMine, we examined gene expression of primary CD4^+^ and CD8^+^ T cells in resting (non-activated) and early activated states of 5 adults. T cell-mediated immunity is the central element of the adaptive immune system and involves constant surveillance from antigen-specific T cells to eliminate infections and malignant cells^21^,^22^,^23^,^24^. T helper cells (CD4^+^) and effector T cells (CD8^+^) play crucial roles in the coordination and function of the adaptive immune system. CD8^+^ T cells are highly effective in direct lysis of pathogen infected cells. CD4^+^ T helper cells produce cytokines that are directly toxic to target cells, stimulate other immune cell lineages, or mobilize powerful inflammatory mechanisms.

Each T cell lineage was sorted from peripheral blood mononuclear cells (PBMCs) at rest or post activation (4 hrs) with PMA/ionomycin in a paired design from N = 5 healthy individuals. We examined gene expression in each T cell subtype using a custom 135-plex NanoString array covering core genes involved in human T cell recognition, survival, migration, adhesion, cytokine/chemokine secretion, differentiation and exhaustion.

Hierarchical clustering and principal components analysis (PCA) showed clear clustering of gene expression profiles by T cell subtype (CD4^+^/CD8^+^) (Fig. 7a and b) and activation status (Fig. 7a and c). These results were supported by multivariate statistics using canonical correspondence analysis (CCA), demonstrating that resting CD4^+^ and CD8^+^ T cells have significantly different gene expression profiles (p = 0.001, CCA). Analysis by CCA further showed that T cell activation significantly altered gene expression (p = 0.001).

#### Resting CD4^+^ and CD8^+^ T cells show markedly different gene expression profiles

As expected, resting CD4^+^ and resting CD8^+^ showed different expression profiles (Fig. 7b) and could be distinguished by differential expression of 67 genes (paired t-test, FDR < 0.05, Table S4), most notably the chemokine CCR4, the costimulatory molecule ICOS, the immune checkpoint receptor CTLA4, the chemokine receptor CCR7 and the transcription factor AHR. Resting CD8^+^ showed higher expression of the classic effector molecules PRF1, GZMA and IFN-γ compared to resting CD4^+^ T cells. Similar to our results obtained by paired t-test, LEfSe identified 63 genes that were differentially expressed between CD4^+^ and CD8^+^ T cells (Table S10).

#### Changes in gene expression after activation with PMA/ionomycin

Post activation, 90 genes were differentially expressed in the CD4^+^ compartment and 84 genes were differentially expressed in the CD8^+^ compartment (FDR < 0.05, paired t-test) (Tables S5, S6). Many genes showed rapid and marked upregulation post activation highlighting how fast transcriptional changes can occur in human lymphocytes. For example, in the CD4^+^ populations, *IL-2* increased from one to over 150,000 molecules per sample within four hours. Across both T cell lineages, *IL-2* was the most statistically robust marker of recent activation (Tables S5, S6 and Figure S5). The largest fold changes in activated CD4^+^ T cells were observed for *IL-17* and *IL-22* (Table S5 and Figure S5a), indicating that mitogen activation strongly drives a Th17 response. Unexpectedly, the “early activation” markers *CD27* and *CD28* were downregulated in post activated CD4^+^ T cells (Figure S5a). The most downregulated gene was *KLF2*, showing abolished expression upon activation (Table S5 and Figure S5a). KLF2 is involved in T cell trafficking and maintaining quiescence.

In the CD8^+^ T cell subset, the top three most highly expressed cytokines post activation were the classic Th1 effector molecules *IL-2, IFN-γ* and *TNF* (Table S6 and Figure S5b). Interestingly, post activated CD8^+^ showed no increase in any effector molecule measured, including *GZMA/B/H/K/M/Y*. Some effector molecules even decreased (Table S6). This is in contrast to CD4^+^ T cells which showed marked increase of *GZMB* post activation (Figure S6). Similar results were obtained using Bayesian t-test (data not shown).

Similar to our results obtained by paired t-test, LEfSe identified an association between the activation status of CD4^+^ and CD8^+^ and the expression of 87 and 91 genes, respectively (Tables S11 and S12).

#### CD4^+^ and CD8^+^ T cells show similar changes in gene expression post activation

Unexpectedly, CD4^+^ and CD8^+^ showed highly similar expression profiles post activation and 73 genes exhibited the same directional expression change across both subsets. This common activation footprint was reflected in a joined cluster of activated CD4^+^ and CD8^+^ lineages in a PCA analysis (Fig. 7c) and suggests the existence of some hardwired activation programs encoded within functionally diverse immune lineages. However, 22 genes showed differential expression in only one of the CD4^+^ and CD8^+^ subtypes (Tables S7, S8).

#### Gene expression profiles highly predictive of activation status

Network analysis of either CD4^+^ or CD8^+^ gene expression profiles resulted in two distinct, highly correlated gene clusters, representing genes with high expression in activated or resting T cells, respectively (Fig. 7e). Gene expression profiles were highly predictive of T cell activation status. A support vector machine (SVM) trained on the gene expression profiles of either CD4^+^ or CD8^+^ and evaluated by leave-one-out cross-validation achieved 100% accuracy in predicting the activation status (resting/activated). An optimal gene signature for predicting T cell activation status was determined by LASSO regularized regression and comprised five genes for the CD4^+^ (Figure S7a) and eight genes for the CD8^+^ compartments (Figure S7b). Two genes were shared across both signatures (STAT5A, IL2), three genes were unique for CD4^+^ (*CD40LG, IRF4, IL10*) and six genes were unique for CD8^+^ (*IL22, GFI1, IL23A, COR6, IL12RB2, ILGA4*).

## Conclusions

GMine provides a comprehensive statistical and visualization toolbox that allows users to rapidly obtain comprehensive analyses from multidimensional biomolecular datasets, without scripting or programming knowledge. Robust and thorough analyses are obtained via a broad range of algorithms for clustering, classification and statistical testing. The software enables lab-based researchers, who may be unfamiliar with advanced statistical software packages, to use these complex types of analyses routinely in their work in order to gain a more comprehensive understanding of their data.

Our re-analysis of *P. falciparum* protein array data replicated previous results, demonstrating that GMine facilitates robust analysis of complex biomolecular datasets. In accordance with Crompton *et al*.^15^ we found that antibody responses to *Plasmodium* proteins are associated with time relative to malaria season, subject age and protection from malaria. Consistent with the previous study, we observed that susceptible children have strong reactivity to some proteins, but protected children tend to have a significantly higher breadth of response. These results are consistent with other reports indicating that the breadth of antibody response to malaria proteins rather than strong reactivity to individual proteins is critical for developing immunity to malaria^25^,^26^,^27^. Antibody response to the malaria vaccine candidates CSP, LSA-3, AMA-1, MSP-1 and MSP-2 did not discriminate between protected and susceptible children in agreement with the previous study^15^, as well as with results of clinical trials of candidate AMA-1^28^ and MSP-1^29^ vaccines.

While Crompton *et al*. focused on individual antigens, we extended this study by analyzing antibody reactivity profiles and antibody responses against combinations of parasite antigens. We demonstrated that children who were protected or susceptible to malaria differed in their global antibody reactivity to *P. falciparum* proteins. Antibody profiles of protected and susceptible children formed distinct clusters when analyzed by hierarchical clustering and were predictive of immunity to malaria when using a SVM classifier. Our results further extend previous findings by identifying an antibody signature that dramatically improves the power to discriminate protected from susceptible children as compared with antibody responses to single antigens. These findings support the hypothesis that immunity to malaria is a higher-order phenomenon related to a combination of antibody reactivity rather than to single antibody reactions, consistent with our previous observations in experimentally immunized populations^6^.

We further extended the previous report by showing that antibody reactivity profiles are associated with age and time during the malaria season on a proteome-wide level, as evident by our cluster analysis. Finally, multivariate analysis in GMine found new positive correlations between antibody reactivity against *Plasmodium* proteins and concurrent parasitemia and day until first febrile malaria episode. Gender and hemoglobin type were not associated with antibody response, consistent with previous studies^30^.

We also used GMine to study the expression of 135 key immune-related genes in CD4^+^ and CD8^+^ T cells in the resting and post activation state. The immunomonitoring of these core immune cell subsets has led to the discovery of many biological correlates of disease. A more complete understanding of T cell function will lead to new avenues for immunotherapeutic development. Previous studies have examined changes in human naïve and memory CD4^+^ T cell phenotypes following mitogen activation using microarrays and found 100–300 genes differentially expressed between states^31^,^32^,^33^. Our study establishes proof-of-concept for the potential of GMine to dramatically advance this or similar fields, taking advantage of the new direct digital NanoString technology which is free from the inherent biases of PCR. Using a custom 135 gene array designed for the sensitive and precise profiling of key immune-related genes, GMine analyses showed a digital view of T cell gene expression (Fig. 7). Second, using GMine, we found that the gene expression profiles of CD8^+^ and CD4^+^ T cells are unique at rest but very similar post activation. The latter was a surprising finding and suggests the existence of a common “hardwired” activation system across many highly diverse human T cell lineages. To our knowledge this has not been described or investigated previously; with prior studies investigating only a single lineage. to our knowledge, previous studies investigated only a single lineage^31^,^32^,^33^,^34^. The rapid post activation phenotype comprised a cytotoxic gene signature across both CD8^+^ and CD4^+^ T cells. Although not widely appreciated, “helper” CD4^+^ T cells have the potential for cytotoxic activity through the rapid production of effector molecules such as granzymes and FasL^35^. Our findings highlight the extreme plasticity inbuilt in T cell biology and function.

Our results demonstrate that GMine provides a new framework for analyzing high-throughput omics’ data and that robust and comprehensive analysis can rapidly be obtained using a wide range of advanced data-mining techniques.

## Methods

### GMine Implementation

The web-frontend is implemented in Java using the JavaServer Faces architecture and the backend is implemented in Perl and the R statistical programming environment using existing packages and newly developed modules. The software is running on a virtual server of the National eResearch Collaboration Tools and Resources (Nectar) cloud. A wiki page providing extensive help and a tutorial, has been created using the MediaWiki package.

Importance of features selected by feature selection is characterized by Akaike information criterion (AIC) of the model if the feature was dropped from the model (step-wise regression), absolute of the t-statistic (LASSO regression), or random permutation (random forest). To generate correlation networks, first associations between features are computed using the Pearson’s correlation index or Spearman’s rho and used to ordinate nodes in a two dimensional plot by principal coordinates analysis (PCoA). In this way, correlating nodes are placed in close proximity and anti-correlating nodes are placed at distant locations in the network. Correlating nodes are then connected with edges.

### Malaria Whole-Proteome Microarray Data

We re-examined our previously published protein microarray data generated in a prospective study investigating host immune response to the malaria parasite *Plasmodium falciparum*^15^. Crompton *et al*. used a protein microarray consisting of 2,320 probes (representing ~23% of the *P. falciparum* proteome) to profile host immune response (IgG) against malaria parasite proteins. The cohort comprised 225 individuals from Kambila, Mali. Plasma samples were collected from each individual before and after the 8-month malaria season. Individuals were classified as protected (no malaria episode during the 8 months study) or susceptible (≥1 malaria episodes). In our reanalysis we included the subset of 194 individuals for whom samples were available at both time points (before and after the malaria season). In accordance with the previous study, we assigned individuals to two age groups: age 2–10 years (n = 157) and 18–25 years (n = 37). Our analysis included antibody profiles of 388 serum samples (2 × 194).

#### Data preprocessing

Before re-analyzing the data in GMine, we first background corrected signal intensities and identified seropositive proteins. Protein microarray raw signal intensities (SI) were corrected for spot-specific background using the Axon GenePix Pro 7 software (Molecular Devices, Sunnyvale, CA). Mean intensity of the No-DNA negative control spots from each sample was subtracted from all individual target protein fragment spots. Negative or zero values after background subtraction were assigned a net value of 0. Background corrected data were normalized by log2-transformation. Proteins were considered seropositive for antibody response if the signal intensity was greater than the mean signal intensity of no-DNA negative controls plus 1.5 times the standard deviation (SD). Only seropositive proteins were analyzed further.

#### Analysis in GMine

For the re-analysis of *Plasmodium* antibody profiles in GMine, we used the normalized antibody signal intensities against the 491 seropositive protein fragments. Two samples were included for each of the 194 individuals, collected before (May) and after (December) the malaria season, resulting in a total of 388 samples. The following clinical variables were included: subject age (assigned to age groups 2–4/5–7/8–10/18–25 years), time of sample collection (before/after the malaria season), parasitemia (microscopically detectable parasite [yes/no]), hemoglobin type (AA, AC, CC), gender, incidence of malaria (number of clinical malaria episode during 8 month study period), and days until the first malaria episode after the sample collection in May [range: 29 days to 242 days (no episodes)].

Antibody profiles were correlated using Pearson’s correlation. Difference in average antibody signal intensities between age groups were tested by ANOVA. Antibody signal intensities before and after the malaria season were compared by paired t- test. The 388 antibody profiles were associated with multiple explanatory variables by the multivariate statistical methods redundancy analysis (RDA) and canonical correspondence analysis (CCA). In this analysis age, time of the malaria season, parasitemia, hemoglobin type, gender, days until the first malaria episode and incidence of malaria were included as explanatory variables.

A subset of 48 children aged 8 to 10 years was evaluated for association between antibody response and protection against clinical malaria. Children were defined as *protected* if they did not experience clinical malaria episode during the 8-month study period (n = 19) and *susceptible* otherwise (n = 29 children). Difference in average signal intensity (magnitude of response) and breadth of response (number of seropositive proteins detected in each individual) between protected and susceptible children were compared by t-test.

Multivariate analysis PCA, PCoA, RDA and CCA were used to assess the association between antibody signal intensities before the malaria season in May and subsequent protection against clinical malaria during the 8-month malaria season. CCA and RDA were run on antibody signal intensities including protection status (protected/susceptible), parasitemia and hemoglobin type as explanatory variables.

The association between antibody response to individual *Plasmodium* proteins and protection from clinical malaria was estimated by linear regression including age as covariate. The regression models had the form: antibody signal intensity = protection status (protected/susceptible) plus age. Antibody signal intensities before the malaria season between protected and susceptible children were compared by t-test. Area under the curve (AUC) was used to measure the power to discriminate between protected and susceptible children.

A support vector machine (SVM) was trained on the antibody profiles measured in May to discriminate between susceptible and protected children. Classification accuracy was evaluated by leave-one-out cross-validation. A forward stepwise logistic regression model was applied to identify the minimal subset of *Plasmodium* proteins associated with protection from clinical malaria. Protection status (protected/susceptible) was included as dependent variable and protein specific antibody signal intensities as explanatory variables. The quality of the final model including all selected proteins was evaluated by AUC and Akaike information criterion (AIC). P-values were adjusted for multiple testing by the False Discovery Rate (FDR) and Bonferroni correction.

### Primary T Cell Activation and Nanostring Profiling

Gene expression changes in primary T cells lineages were explored using the NanoString technology in order to determine key pathways differentially used between rest and stimulation state. We designed a custom 135-plex nCounter custom codeset around genes encoding for T cell recognition, including cytokines/chemokines, cytokine receptors/chemokine receptors, effector molecules, subset markers, surface receptors, transcription factors, early activation markers, checkpoint markers and signaling molecules. Genes were selected based on published human T cell microarray data and current knowledge in infectious disease, autoimmunity and cancer immunobiology. Peripheral blood mononuclear cells (PBMCs) were derived from 5 healthy genetically unrelated individuals. Experimental protocols were approved by the QIMR Berghofer Medical Research Institute Human Research Ethics Committee and the methods were carried out in accordance with the relevant guidelines and regulations. All samples were obtained with written informed consent. PBMC were isolated by Ficoll-Paque PLUS (GE Healthcare) density gradient centrifugation and cryopreserved in R10 medium (RPMI-1640 containing 10% FBS) supplemented with 10% DMSO (Sigma-Aldrich). PBMCs were then split and left to rest or activated with 1X phorbol12-myristate13-acetate (PMA)/ionomycin (Jomar Life Research) for 4 hrs in R10. Cells were washed twice with PBS (Life Technologies) and stained with LIVE/DEAD^®^ Fixable Aqua and Violet Dead Cell Stain Kits (Life Technologies) and anti-CD14/CD16/CD19/CD3/CD4/CD8 (BioLegend). CD4^+^ (CD14^−^CD16^−^CD19^−^CD3^+^ CD4^+^ CD8^−^) and CD8^+^ (CD14^−^CD16^−^CD19^−^CD3^+^ CD4^−^CD8^+^ ) T cell populations from both activated and resting subsets were then sorted using an Aria IIIu (BD Biosciences) to > 99% purity. Total RNA from each population was extracted using RNAzol RT (Astral Scientific), isopropanol (Sigma Aldrich) and ethanol (Sigma Aldrich). Overnight nCounter codeset hybridisation was performed as per manufacturer’s instruction using 100 ng of sample RNA quantified by Nanodrop 1000 (Thermo Scientific).

#### Data preprocessing

Samples were processed using the NanoString GEN2 Prep Station and data acquired using the nCounter Digital Analyser (NanoString Technologies). Quality control and normalization was performed using nSolver (NanoString Technologies) as per the recommended settings. Quality control parameters included background, positive control and housekeeping normalization using spiked positive and negative controls, as well as five housekeeping genes (ACTB, B2M, GAPDH, HPRT1 and RPLP0). Normalization was performed on CD4^+^ and CD8^+^ T cell populations separately.

#### Analysis in GMine

Normalized data were log2-transformed in GMine. Gene expression profiles were associated with the T cell subtype (CD4^+^/CD8^+^) and with T cell activation status (activated/resting) using the multivariate statistical method canonical correspondence analysis (CCA). T cell subtype and T cell activation state were included as explanatory variables. Changes in expression of individual genes between activated and non-activated (resting) cells and between CD4^+^ and CD8^+^ were identified by paired t-test. P-values were adjusted for multiple testing by the False Discovery Rate (FDR) and Bonferroni correction. Gene expression profiles were correlated by Pearson’s correlation. A support vector machine (SVM) was trained on the gene expression profiles of either CD4^+^ or CD8^+^ to discriminate between activated and resting cell population and evaluated by leave-one-out cross-validation. Gene signatures for predicting T cell activation status were determined by LASSO regularized regression of the differentially expressed genes identified by the paired t-test. The quality of the final model including all selected genes was evaluated by AUC and Akaike information criterion (AIC).

## Additional Information

**How to cite this article**: Proietti, C. *et al*. Mining, visualizing and comparing multidimensional biomolecular data using the Genomics Data Miner (GMine) Web-Server. *Sci. Rep.* **6**, 38178; doi: 10.1038/srep38178 (2016).

**Publisher's note:** Springer Nature remains neutral with regard to jurisdictional claims in published maps and institutional affiliations.

## Supplementary Material

Supplementary Figures

Supplementary Tables

## Figures and Tables

**Table 1:** Overview of graphical and statistical methods provided by GMine.

Clustering	Description
Method	
Network analysis	Network of correlating features
PCoA, PCA, DCA, NMDS	Unsupervised ordination methods used for data clustering and the identification of outliers
LLE, t-SNE	Non-linear methods for dimensionality reduction used to present data in a two dimensional plot
Heatmaps	Heatmap of measurements. Data points are ordered by hierarchical clustering. Identify outliers, sample clusters and associations with explanatory variables.
Hierarchical clustering	Identification of outliers and sample clusters
Self-organizing map	Unsupervised data clustering
***Multivariate analysis***	
***Method***	***Description***
Partial least squares regression (PLS)	Multivariate method used to identify features associated with multiple explanatory variables
Redundancy analysis(RDA), canonical correspondence analysis (CCA)	Supervised multivariate method for identifying significant associations between measurements and multiple explanatory variables
***Identification of significant different features***	
***Method***	***Description***
	Identify features differentially abundant between two or more biological conditions
DESeq2, ANCOM, ALDEx2	Analysis of counts data (e.g. RNA-seq or metagenomic data). Identify features differentially abundant between two biological conditions.
Multiple linear regression	Identify associations between individual features and multiple explanatory variables
Linear mixed effect regression	Analysis of data with repeated measures, e.g. longitudinal data
***Classification and feature selection***	
***Method***	***Description***
Support Vector Machine (SVM)	Examine if analyzed data is predictive of an outcome of interest
Step-wise regression, LASSO regularized regression, random forest	Feature selection identifying a subset of relevant features predictive of an outcome of interest
LDA Effect Size (LEfSe)	Identifies features (e.g. genes, pathways, or proteins) characterizing the differences between two or more biological conditions
***Other features***	
***Method***	***Description***
Boxplot, stripchart, barchart, violin plot	Quantitative visualization of measurements
Square root, log, asinh, asin(sqrt) transformation; quantile normalization, variance stabilization and calibration for microarray data (VSN), centered log ratio	Data transformation and normalization to render data suitable for analysis by standard statistical procedures
Factor analysis	Data reduction as a pre-processing step

**Table 2:** Overview of GMine analysis tabs.

Method	Description
Upload	Data upload
Data	Information on uploaded data
QViz	Quantitative visualization of data as heatmap, barchart or bubble plots. Measurements are visualized for *individual samples*.
GroupPlots	Comparison of measurements across multiple biological conditions (e.g. using ANOVA or non-parametric rank tests). Features that are significantly differentially distributed are presented as boxplots or barcharts.
Features	Quantitative visualization of measurements obtained for individual features.
Stats	Comparison of measurements across multiple biological conditions (e.g. using ANOVA, non-parametric rank tests, Bayesian ANOVA or DESeq2). P-values are adjusted for multiple testing and results are presented as table.
Multivariate	Multivariate data analysis. Various methods are available for data ordination (e.g. PCA, PCoA, NMDS) and multivariate statistical testing (e.g. CCA, RDA).
Feat. Select	Feature selection using stepwise regression, LASSO regression, random forest or LEfSe
Network	Network analysis and clustering using self organizing maps
Biomarker	Identification of biomarker candidates. Biomarkers are characterized by Area Under the Curve (AUC), fold change and delta (difference in mean in units of standard deviation). Results are presented as table and forest plot.
Regression	Identification of associations between measurements and multiple explanatory variables using multivariable regression techniques
Rep. Measures	Analysis of experimental data from repeated measures designs using mixed effect regression models. This technique can distinguish between group-specific effects and subject or cage-specific effects.
Paired	Analysis of paired data using paired t-test or paired rank test
Norm	Visualize the effect of different data transformation and data normalization methods
FactorAnalysis	Factor analysis to reduce data dimensionality and remove redundant features
