# Supplementary material for: Mining, visualizing and comparing multidimensional biomolecular data using the Genomics Data Miner (GMine) Web-Server
Source: Sci Rep. 2016 Dec 6;6:38178. doi: 10.1038/srep38178 (PMC5138638; doi:10.1038/srep38178)

Mining, visualizing and comparing multidimensional biomolecular data using the Genomics Data Miner (GMine) Web-Server.

Carla Proietti*, Martha Zakrzewski*, Thomas Watkins, Bernard Berger, Shihab Hasan, Champa N. Ratnatunga, Marie-Jo Brion, Peter D. Crompton, John Miles, Denise Doolan, Lutz Krause

SUPPLEMENTARY FIGURES

Figure S1: Normalization in GMine. The upper boxplot shows raw measurements, the second boxplot shows transformed data. Additionally, raw measurements are plotted versus transformed measurements. Shown are antibody signal intensities of 491 seropositive proteins before and after VSN transformation for 19 protected children (blue boxplot) and the 29 susceptible children (red boxplot).

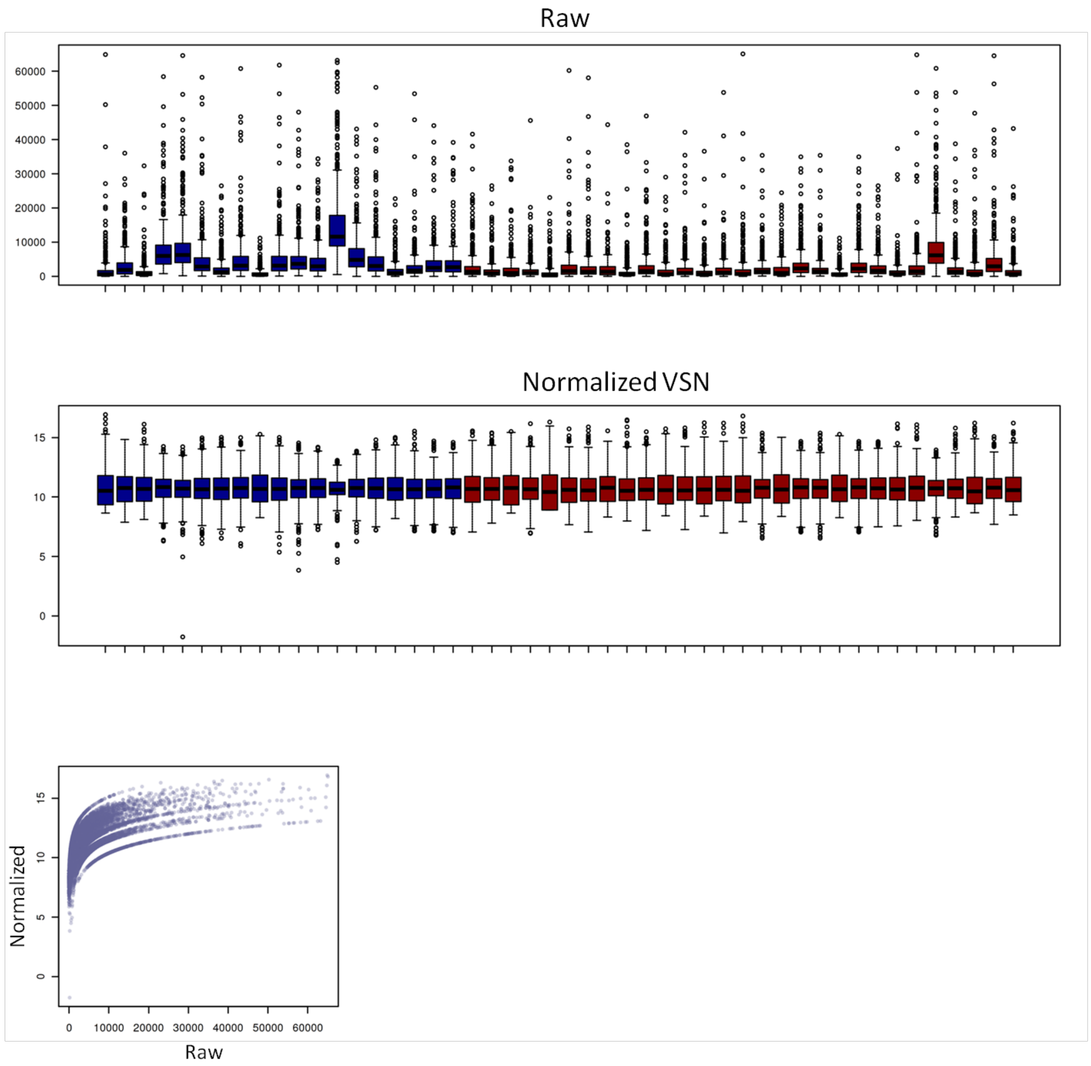

Figure S2. Multivariate analyses of antibody signal intensities against *P. falciparum* proteins in respect to age, time of the malaria season, concurrent parasitemia, gender and hemoglobin type (n=194 individuals). (a,c) Principal components analysis (PCA) of antibody signal intensity profiles (388 samples from 194 individuals). Subjects from different age groups and samples collected before or after the malaria season form concentric clusters. (b,d) Redundancy analysis (RDA) was run on the antibody profiles against 491 seropositive proteins including age, time of the malaria season, concurrent parasitemia, gender and hemoglobin type as explanatory variables. Samples clustered by age group and time of the malaria season (before/after). Age, time of the malaria season and concurrent parasitemia were significantly associated with antibody profiles (p<0.001), while gender (p=0.205) and hemoglobin type (p=0.232) were not.

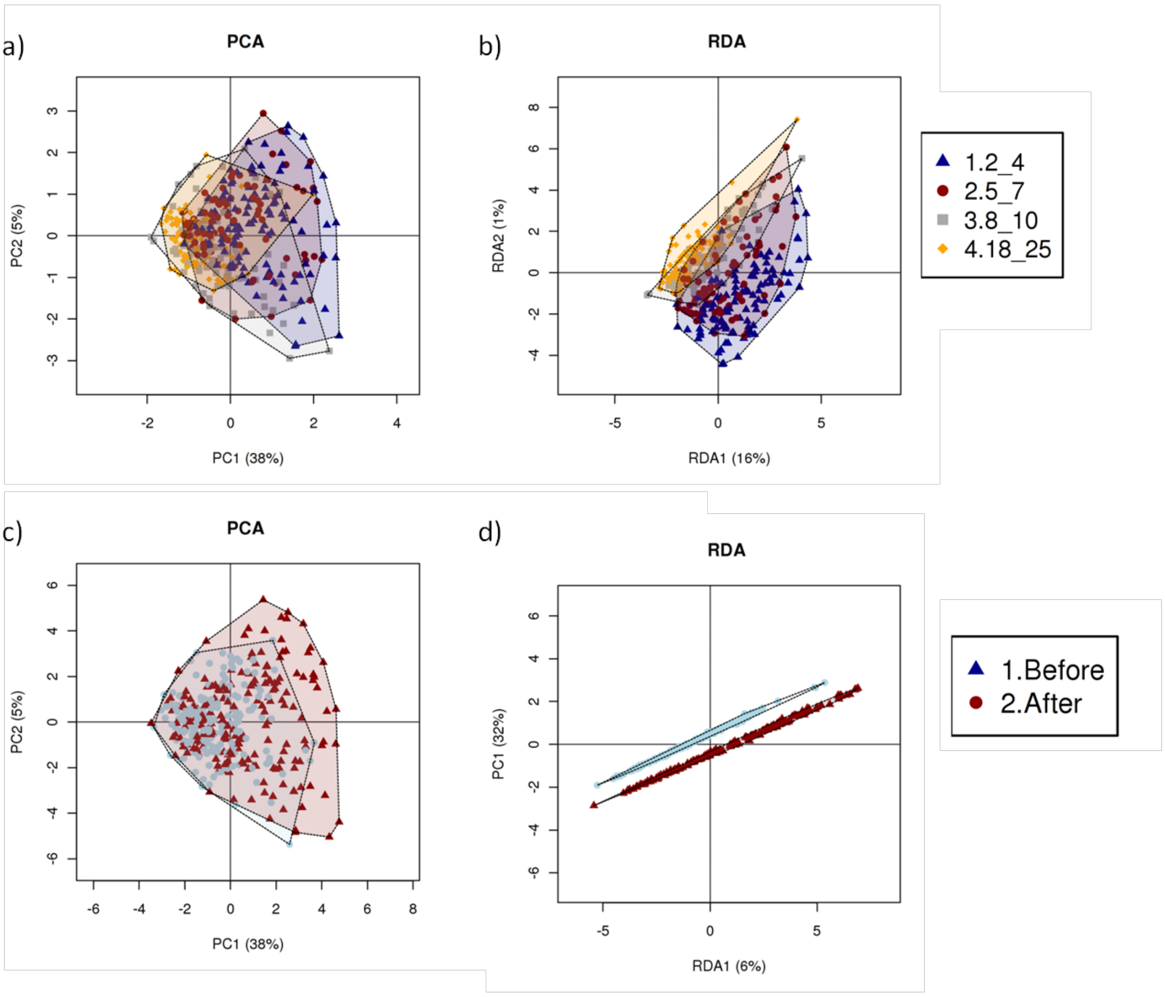

Figure S3. Multivariate analyses of the association between antibody signal intensities (SI) and protection against clinical malaria, concurrent parasitemia, gender and hemoglobin type. Children aged 8-10 years of age (n=48) were defined “protected” if they did not experience clinical malaria episode during the 8-month study period (n=19) and “susceptible” if they did experience ≥1 malaria episodes during the 8-month study period (n=29 children). Antibody response was measured at the beginning of the 8-month study period before the malaria season. a) Principal components analysis (PCA) and b) redundancy analysis (RDA) of the antibody signal intensity profiles. RDA included protection from malaria (protected/ susceptible), gender, concurrent parasitemia and hemoglobin type as explanatory variables. Immunity to malaria was significantly associated with antibody response (RDA p=0.003), while gender (RDA p=0.460), concurrent parasitemia (RDA p=0.507) and hemoglobin type (RDA p=0.438) were not.

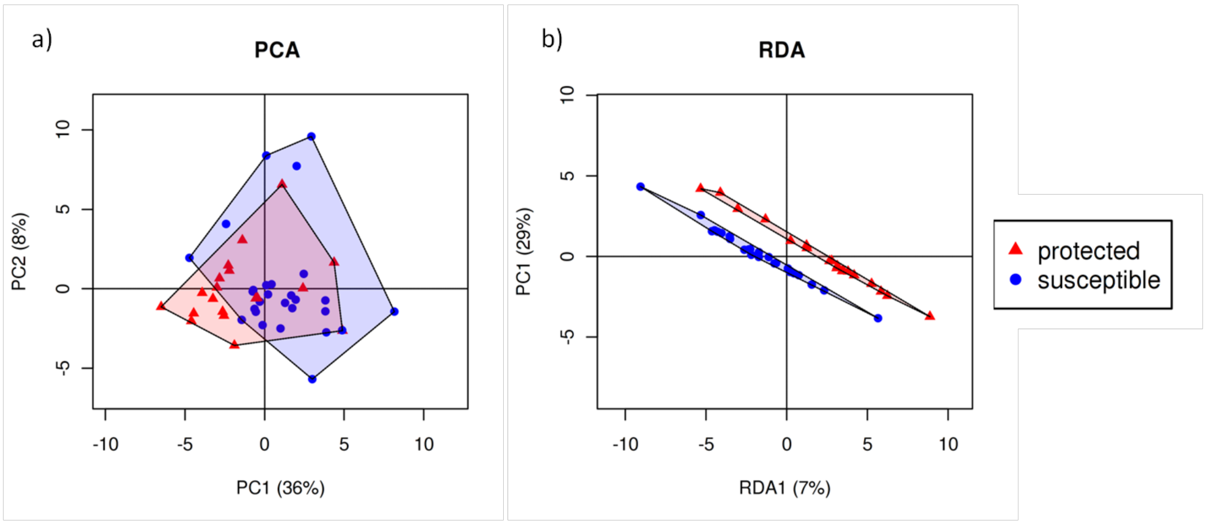

Figure S4. Stepwise regression (forward and backward) applied on the 62 proteins significantly associated with protection from malaria. Regression identified the optimal protein signature for predicting protection against clinical malaria. A six protein signatuare was identified. The AUC of the model was 1. (a) The six proteins selected by stepwise regression are presented as bar charts, where bars depict the importance of each protein (AIC of the model if the protein was dropped from the model). (b) A linear regression model including all six selected proteins achieved perfect classification (AUC of 1).

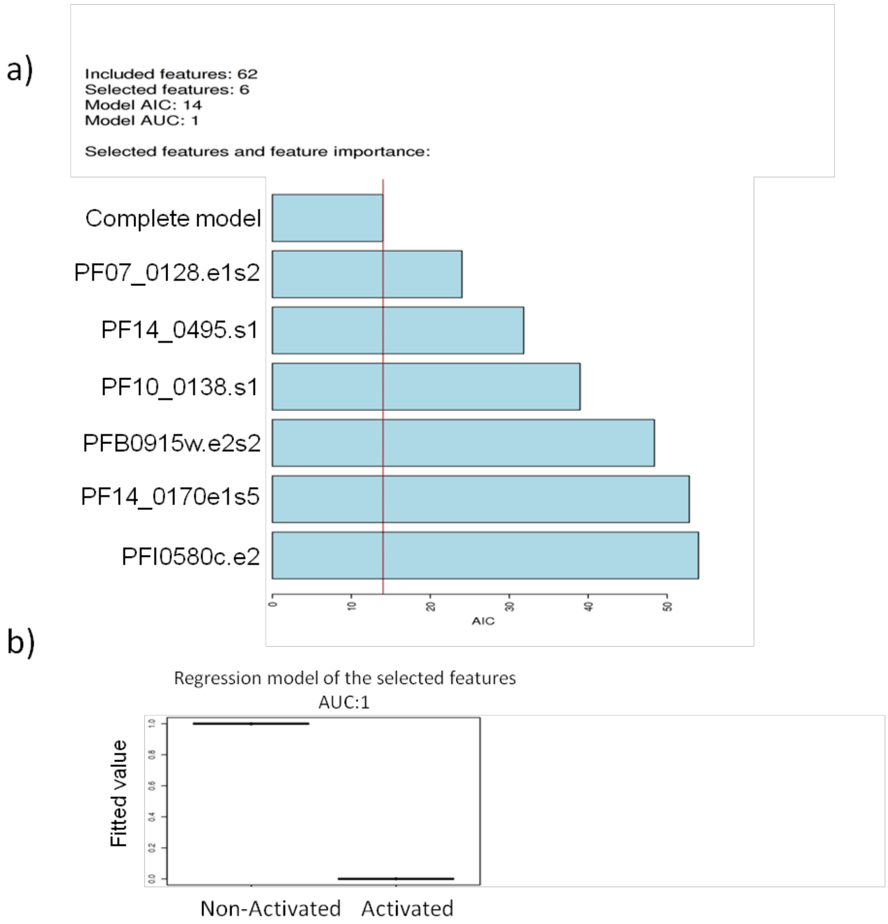

Figure S5. Expression of selected genes *IL2, IL17A, IL22, CD27, CD28* in resting and activated CD4+ (a) and CD8+ (b) T cells. Significance of differences was tested by Wilcoxon rank test.

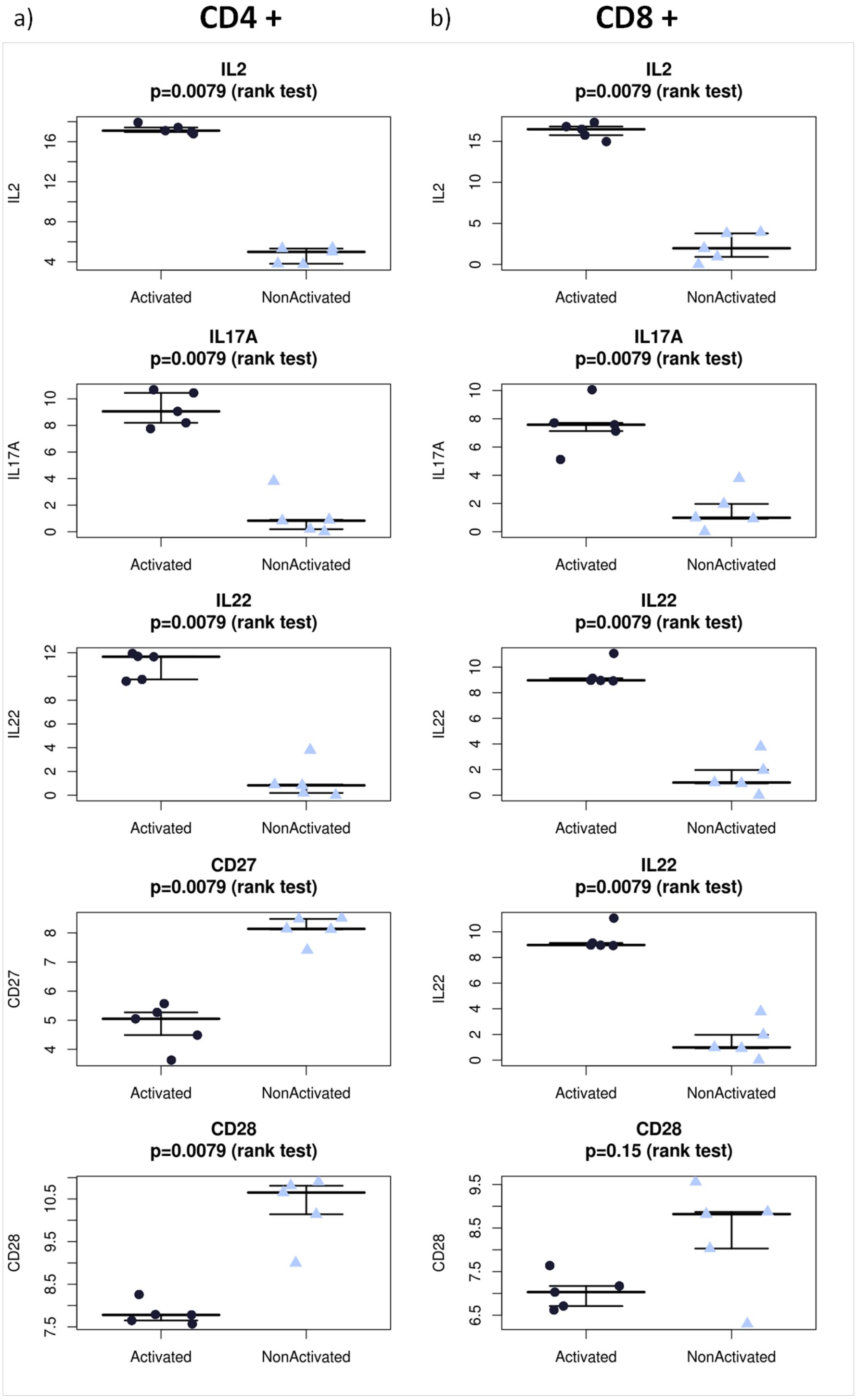

Figure S6. Expression of the *GZMB* gene in resting and activated $CD4^+$ (a) and $CD8^+$ (b) T cells.

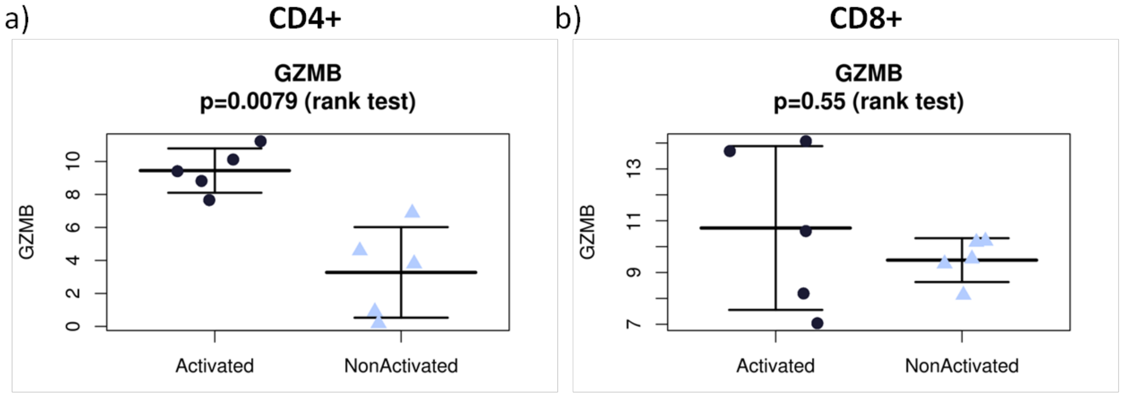

Figure S7: Optimal gene signature for predicting T cell activation status as determined by LASSO regularized regression for CD4$^+$ (a) and CD8$^+$ (b) subtypes. LASSO regularized regression was applied on the 90 genes differentially expressed between activated and non-activated cell in CD4$^+$ and on the 84 genes differentially expressed between activated and non-activated cell in CD8$^+$. A five-gene signature and an eight-gene signature were identified for CD4$^+$ and CD8$^+$, respectively. The AUC of both models was 1. (a,b) Genes selected by LASSO are presented as bar charts, where bars depict the importance of each gene (absolute of t-statistics). (c,d) Linear regression model regressing T cell activation status on selected gene signature achieves perfect classification (AUC of 1) for CD4$^+$ (c) and CD8$^+$ (d), respectively.

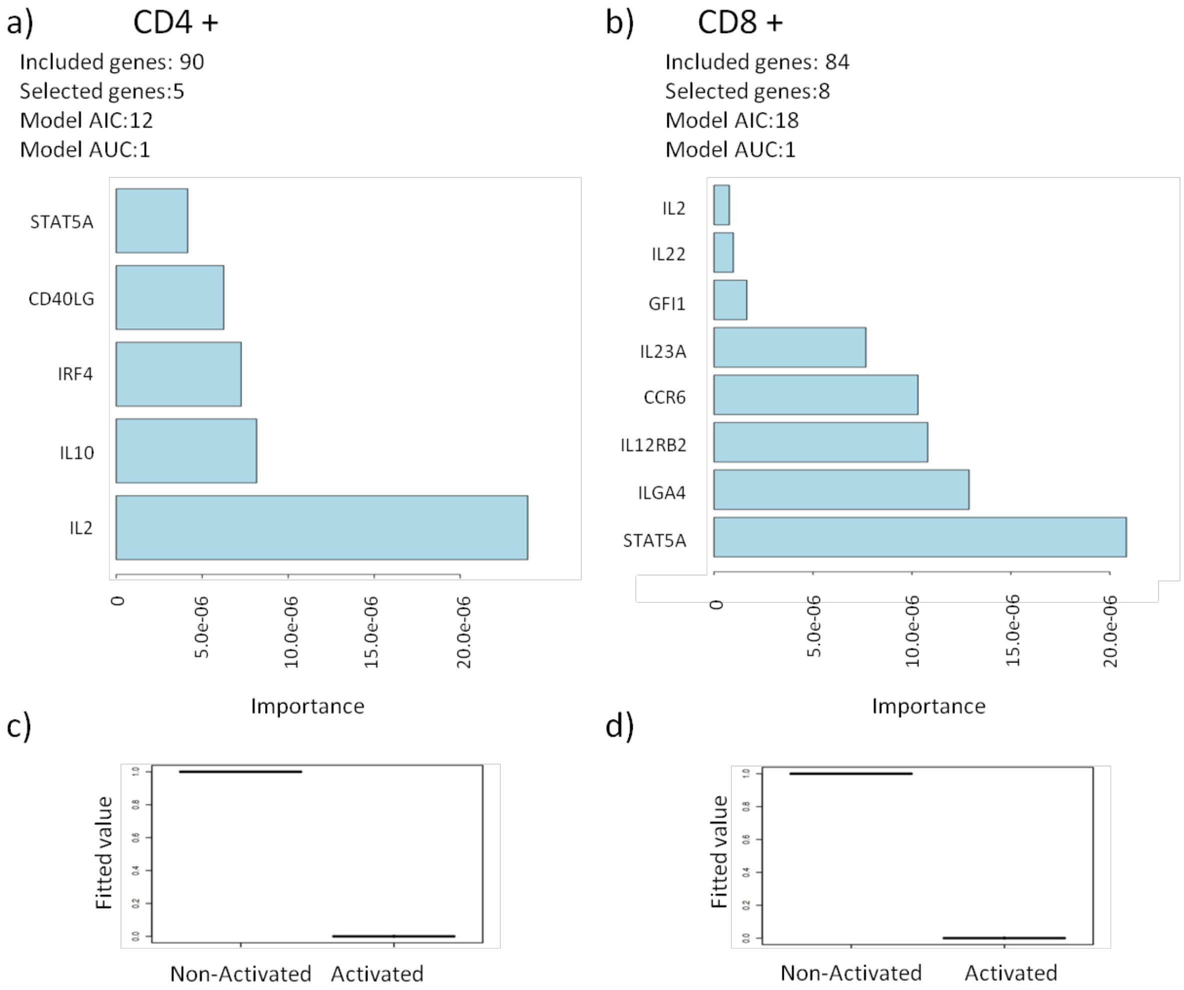

Supplement: Supplementary Figures [file srep38178-s1.pdf]
